# Heart Rate Variability for Early Detection of Cardiac Iron Deposition in Patients with Transfusion-Dependent Thalassemia

**DOI:** 10.1371/journal.pone.0164300

**Published:** 2016-10-13

**Authors:** Suchaya Silvilairat, Pimlak Charoenkwan, Suwit Saekho, Adisak Tantiworawit, Arintaya Phrommintikul, Somdet Srichairatanakool, Nipon Chattipakorn

**Affiliations:** 1 Department of Pediatrics, Faculty of Medicine, Chiang Mai University, Chiang Mai, Thailand; 2 Department of Radiologic Technology, Faculty of Associated Medical Sciences, Chiang Mai University, Chiang Mai, Thailand; 3 Biomedical Engineering Center, Faculty of Engineering, Chiang Mai University, Chiang Mai, Thailand; 4 Department of Internal Medicine, Faculty of Medicine, Chiang Mai University, Chiang Mai, Thailand; 5 Department of Biochemistry, Faculty of Medicine, Chiang Mai University, Chiang Mai, Thailand; 6 Cardiac Electrophysiology Research and Training Center, Faculty of Medicine, Chiang Mai University, Chiang Mai, Thailand; 7 Cardiac Electrophysiology Unit, Department of Physiology, Faculty of Medicine, Chiang Mai University, Chiang Mai, Thailand; 8 Center of Excellence in Cardiac Electrophysiology Research, Chiang Mai University, Chiang Mai, Thailand; Northeastern University, UNITED STATES

## Abstract

**Background:**

Iron overload cardiomyopathy remains the major cause of death in patients with transfusion-dependent thalassemia. Cardiac T2* magnetic resonance imaging is costly yet effective in detecting cardiac iron accumulation in the heart. Heart rate variability (HRV) has been used to evaluate cardiac autonomic function and is depressed in cases of thalassemia. We evaluated whether HRV could be used as an indicator for early identification of cardiac iron deposition.

**Methods:**

One hundred and one patients with transfusion-dependent thalassemia were enrolled in this study. The correlation between recorded HRV and hemoglobin, non-transferrin bound iron (NTBI), serum ferritin and cardiac T2* were evaluated.

**Results:**

The median age was 18 years (range 8–59 years). The patient group with a 5-year mean serum ferritin >5,000 ng/mL included significantly more homozygous β-thalassemia and splenectomized patients, had lower hemoglobin levels, and had more cardiac iron deposit than all other groups. Anemia strongly influenced all domains of HRV. After adjusting for anemia, neither serum ferritin nor NTBI impacted the HRV. However cardiac T2* was an independent predictor of HRV, even after adjusting for anemia. For receiver operative characteristic (ROC) curve analysis of cardiac T2* ≤20 ms, only mean ferritin in the last 12 months and the average of the standard deviation of all R-R intervals for all five-minute segments in the 24-hour recording were predictors for cardiac T2* ≤20 ms, with area under the ROC curve of 0.961 (p<0.0001) and 0.701 (p = 0.05), respectively.

**Conclusions:**

Hemoglobin and cardiac T2* as significant predictors for HRV indicate that anemia and cardiac iron deposition result in cardiac autonomic imbalance. The mean ferritin in the last 12 months could be useful as the best indicator for further evaluation of cardiac risk. The ability of serum ferritin to predict cardiac risk is stronger than observed in other thalassemia cohorts. HRV might be a stronger predictor of cardiac iron in study populations with lower somatic iron burdens and greater prevalence of cardiac iron deposition.

## Introduction

Thalassemia is a hereditary blood disorder caused by a defective synthesis of the globin chains that are the main components of hemoglobin. Transfusion-dependent thalassemia (TDT) is a subset of thalassemia that requires regular red blood cell transfusions for survival. The only curative treatment for TDT is stem cell transplantation, which has limited availability due to a lack of donors. Therefore, the mainstay of long-term management is red blood cell transfusion. However, the resultant iron overload causes severe damage of many organs [1–2]. A serious consequence of iron deposition is cardiotoxicity, the leading cause of death among these patients. The survival of TDT patients is determined by the amount of iron accumulation within the heart [3–4]. Although there are many iron chelators currently available that are effective at removing excess accumulated iron, the mortality of TDT remains high [5–9].

Non-invasive assessments of iron accumulation, including serum ferritin, non-transferrin bound iron (NTBI) and cardiac T2-star (T2*) magnetic resonance imaging (MRI), are used to monitor the amount of iron and to serve as a guide for iron chelation therapy. Cardiac T2* MRI is a non-invasive modality for cardiac iron quantification that is used as a gold standard for early detection of iron overload cardiomyopathy [10–17]. Decreased cardiac T2* is correlated with ventricular dysfunction in TDT [10]. However, cardiac T2* MRI usage is currently limited because of its cost and availability. Therefore, cardiac T2* MRI might not be a practical method for early detection of cardiac iron status in TDT patients in developing countries.

Heart rate variability (HRV) has been used as a prognostic factor for chronic heart failure [18]. HRV is depressed in thalassemia patients [19–20]. HRV has been proposed as a potential indicator of early detection of cardiac siderosis [21–23]. We hypothesize that HRV reflects the severity of iron overload and can serve as an early detection of iron deposits in the heart. As cardiac T2* MRI has recently been used to evaluate the iron accumulation in the heart, the aim of this study is to determine the relationship between HRV and cardiac T2* in a large population of patients with TDT.

## Materials and Methods

One hundred and one patients with TDT at Chiang Mai University Hospital were enrolled in this study. The study protocol was reviewed and approved by the institutional Ethics Committee of the Faculty of Medicine, Chiang Mai University, Chiang Mai, Thailand. All patients, or their legal guardians, gave written informed consent for research participation. Patients older than eight years of age with thalassemia who received blood transfusions of more than 12 units per year were included in the study. We excluded patients with a contraindication to MRI, clinical symptoms of overt congestive heart failure, and patients using medications that could affect the cardiac autonomic balance. Ninety-six patients received iron chelation therapy (deferoxamine, deferasirox and deferiprone). Only five patients had not taken iron chelation therapy due to low levels of serum ferritin. The patients who underwent deferiprone therapy were requested to stop chelation for 48 hours before blood collection. Laboratory profiles included hemoglobin, serum ferritin and NTBI. Echocardiography, 24-hour Holter monitoring for HRV analysis and cardiac T2* MRI were performed in all patients upon entry. Fifteen volunteers entering as the control group underwent cardiac T2* MRI and 24-hour Holter monitoring for HRV analysis.

### Laboratory profiles

Mean hemoglobin during the last 12 months and mean and maximum ferritin during the past 5 years were studied. Serum NTBI was measured by using nitrilotriacetic acid (NTA) chelation and a high-performance liquid chromatography (HPLC) method [24–25]. In assay, 450-μL serum was incubated at room temperature for one hour with 50 μL of 800-mM NTA with a final concentration of 80 mM and further incubated for 30 minutes to produce a ferric-nitrilotriacetate complex, Fe^3+^-(NTA)_2_. After incubation, the Fe^3+^-(NTA)_2_ was separated from the serum proteins by centrifuging through the membrane filter (30-kDa cut-off, polysulfone type, 0.5 mL capacity) at 12,000 g, 15°C for 45 minutes. The ultra-filtrate was injected into a non-metallic 50 μL loop and analyzed by using HPLC. The analysis was fractionated into a glass column (ChromSep-ODS1, 100 mm x 3.0 mm, 5 μm) and eluted with mobile-phase solvent (3 mM CP22 in 19% acetronitrile buffered with 5 mM MOPS pH 7.0). Fe^3+^-(NTA)_2_ was fractionated on the column, then derivatized with CP22 to form a Fe^3+^-(CP22)_3_ complex, which was detected at 450 nm with the SpecMonitor^®^ 2300 flow-cell detector (LDC Milton-Roy Inc., FL, USA). The NTBI peak height was integrated and recorded for determination of NTBI concentration from the calibration curve. The calibration curve was produced by plotting peak height values on the y-axis against iron concentration on the x-axis. A linear regression line was used to calculate the plasma NTBI concentration.

### Heart rate variability analysis

A 24-hour Holter monitoring system, GE Seer Light Extension (GE Medical Systems, Freiburg, Germany), was utilized for HRV analysis. The recordings were reviewed and then the HRV was calculated by the analysis software MARS. Then, a manual check was performed by the investigator to remove possible artifacts being counted as QRS complexes, which took about 20–30 minutes per case. HRV analysis included frequency domains and time domains. Frequency-domains of HRV parameters were low-frequency (LF) power (about 0.04–0.15 Hz), high-frequency (HF) power (about 0.15–0.4 Hz) and the LF⁄HF ratio. Time domains of HRV parameters were the standard deviation of all normal sinus R-R intervals in the entire 24-hour recording (SDNN), the standard deviation of all average normal sinus R-R intervals for all five-minute segments in the 24-hour recording (SDANN), the average of the standard deviation of all R-R intervals for all five-minute segments in the 24-hour recording (ASDNN) and the root mean square of the mean of the squared differences of two consecutive R-R intervals in the 24-hour recording (rMSSD) [23].

### Echocardiography

Echocardiographic data were obtained by using the Philips iE33 system (Philips Healthcare, Bothell, WA, USA). A complete two-dimensional, M-mode, Doppler (pulsed wave, continuous wave and color) echocardiography was performed. Echocardiographic parameters included left ventricular end diastolic dimension, left ventricular end systolic dimension, left ventricular fractional shortening and left ventricular ejection fraction (LVEF). LVEF was measured by using M-mode and modified Simpson’s method.

### Cardiac T2-star magnetic resonance

All patients underwent a cardiac T2* MRI by using a 1.5 Tesla MRI scanner (Philips Achieva, the Netherlands) with a sense cardiac phase array of a five-element coil, or SENSE XL Torso 16-element coil, as was used in previous studies [26]. Cardiac T2* was measured from a single short-axis view at the mid-left ventricle with ten echo times (1.70–26.10 ms with an increment of 2.70 ms). The cardiac T2* protocol included a double inversion recovery black blood gradient echo. A multi-echo sequence was applied with a flip angle of 25°, matrix 164x154, FOV 36 cm, TR 28 ms, slice thickness 10 mm, and number of signal averages (NSA) = 1. Data analysis was performed on the workstation with the validating in-house software developed on MATLAB R2014b (Mathworks, Natick, MA, USA).

### Statistical analysis

All statistical calculations were assessed using commercially available software (SPSS version 22, SPSS Inc., Chicago, IL, USA). Continuous data were expressed as median and range. Categorical data were summarized with the number and percentage. The relationships between HRV and hemoglobin, serum ferritin, NTBI and cardiac T2* were evaluated using multiple linear regression analysis. The receiver operative characteristic (ROC) curve was analyzed to determine the relationship between cardiac T2* and HRV. A p-value less than 0.05 was considered statistically significant.

## Results

One hundred and one patients with transfusion-dependent thalassemia were studied (median age 18 years, range 8 to 59 years). Forty-seven patients (47%) were male. Forty-seven patients had homozygous β-thalassemia and 54 patients had Hb E/β-thalassemia. Sixty-four patients (63%) underwent splenectomy. Severity of iron overload was classified by the mean serum ferritin level during the past 5 years, and patients were split into three groups: serum ferritin <2,500 ng/mL, 2,500–5,000 ng/mL and >5,000 ng/mL [27]. Baseline clinical and laboratory characteristics according to the severity of iron overload are summarized in Table 1. Chelation therapy according to the mean serum ferritin during the past 5 years is summarized in S1 Table. The patient group with a mean serum ferritin during the past 5 years >5,000 ng/mL consisted of significantly more homozygous β-thalassemia and splenectomized patients, had lower hemoglobin level, and had more cardiac iron deposits (as indicated by lower duration of the MRI T2*) than the group with a mean serum ferritin <2,500 ng/mL. The age, sex, NTBI and LVEF were not statistically different among these three groups. All patients had normal systolic left ventricular function (LVEF 69±6%). The six patients with cardiac T2* ≤20 ms had mean ferritin in the last 12 months with median of 3,767 (range 2,800–9,006 ng/mL), mean ferritin during the past 5 years with median of 5,141 (range 3,852–10,182 ng/mL) and median NTBI of 2.01 (range 1.08–15.39 μM). There was good correlation between mean ferritin in the last 12 months and cardiac T2* (r = -0.523, p<0.0001). However, no correlation was determined between NTBI and mean ferritin in the last 12 months (r = -0.158, p = 0.130) and between NTBI and cardiac T2* (r = 0.029, p = 0.782).

**Table 1:** Baseline clinical and laboratory characteristics according to the mean serum ferritin during the past 5 years.

	Serum ferritin <2,500 ng/mL (N = 64)	Serum ferritin 2,500–5,000 ng/mL (N = 26)	Serum ferritin >5,000 ng/mL (N = 11)	P value
Age (years)	16.8 (8.1–59.0)	21.4 (8.7–56.0)	17.0 (12.4–24.2)	0.061
Male (N, %)	30 (47%)	11 (42%)	6 (55%)	0.789
Homozygous β-thalassemia (N, %)	19 (30%)	18 (69%)*	10 (91%)*	<0.0001
Splenectomy (N, %)	34 (53%)	19 (73%)	11 (100%)*#	0.0009
Hemoglobin (g/dL)	8.1 (4.2–9.7)	7.2 (5.9–9.0)*	6.9 (6.1–8.1)*	0.0006
NTBI (μM)	2.9 (0.02–30.4)	4.4 (0.4–12.7)	2.2 (1.1–15.4)	0.469
Cardiac T2* (ms)	35.6 (24.2–46.7)	36.9 (20.9–47.9)	17.2 (4.0–36.8)*#	<0.0001
LVEF (%)	69.5 (55.0–81.2)	69.2 (55.0–78.1)	67.3 (60.1–77.5)	0.870

NTBI, non-transferrin bound iron; LVEF, left ventricular ejection fraction; DFO, deferoxamine; DFX, deferasirox; DFP, deferiprone.

* p<0.05 vs. ferritin <2,500 ng/mL

^#^ p<0.05 vs. ferritin 2,500–5,000 ng/mL

The HRV parameters in both time and frequency domains were stratified by the mean serum ferritin during the past 5 years (<2,500 ng/mL; 2,500–5,000 ng/mL; and >5,000 ng/mL) and are summarized in Table 2. The time domain (rMSSD) of HRV in patients with a mean serum ferritin >5,000 ng/mL was significantly lower than that of those with a mean serum ferritin between 2,500–5,000 ng/mL and <2,500 ng/mL (p = 0.03). The LF/HF ratio of the HRV was significantly higher in patients with a mean serum ferritin >5,000 ng/mL (p = 0.04). However, serum ferritin was not a predictor for the HRV using multiple linear regression analysis.

**Table 2:** Heart rate variability parameters according to the mean serum ferritin during the past 5 years.

	Serum ferritin <2,500 ng/mL (N = 64)	Serum ferritin 2,500–5,000 ng/mL (N = 26)	Serum ferritin >5,000 ng/mL (N = 11)	P value
**Time domain**				
SDNN (ms)	110 (55–178)	108 (65–285)	101 (63–140)	0.313
SDANN (ms)	103 (49–178)	103 (58–322)	86 (62–137)	0.338
ASDNN (ms)	42 (18–95)	41 (22–339)	34 (16–60)	0.287
rMSSD (ms)	27 (9–56)	21 (9–47)*	17 (8–35)*	0.032
**Frequency domain**				
LF (ms^2^)	14.6 (4.2–35.7)	14.6 (6.0–52.3)	13.4 (4.0–23.8)	0.327
HF (ms^2^)	12.5 (2.9–29.8)	9.9 (3.2–27.2)	8.5 (1.9–22.8)	0.139
LF/HF ratio	1.2 (0.9–2.0)	1.3 (0.9–2.2)*	1.4 (1.0–2.2)*	0.044

SDNN, the standard deviation of all normal sinus R-R intervals in the entire 24-hour recording; SDANN, the standard deviation of all average normal sinus R-R intervals for all 5-minute segments in the 24-hour recordings; ASDNN, the average of the standard deviation of all R-R intervals for all 5-minute segments in the 24-hour recordings; rMSSD, the root mean square of the mean of the squared differences of two consecutive R-R intervals in the 24-hour recordings; LF, low frequency power; HF, high frequency power.

* p<0.05 vs. ferritin <2,500 ng/mL

### Relationship between HRV and hemoglobin, serum ferritin, NTBI, and cardiac T2*

**Fig 1:**
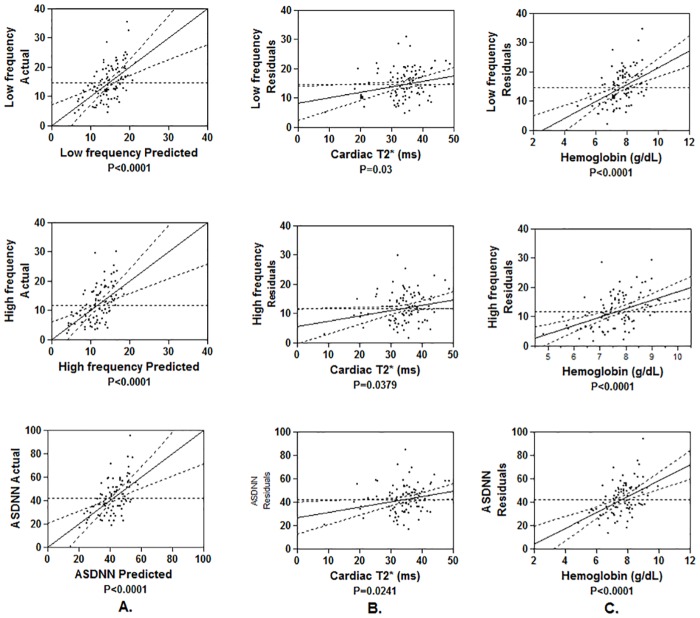
The scatter plots for the correlation between HRV [1) low frequency (upper row), 2) high frequency (middle row) and 3) ASDNN (lower row)] and hemoglobin and cardiac T2*. A: whole model of multiple linear regression analysis; B: the correlation between HRV and cardiac T2*; C: the correlation between HRV and hemoglobin. ASDNN, the average of the standard deviation of all R-R intervals for all 5-minute segments in the 24-hour recordings.

**Fig 2:**
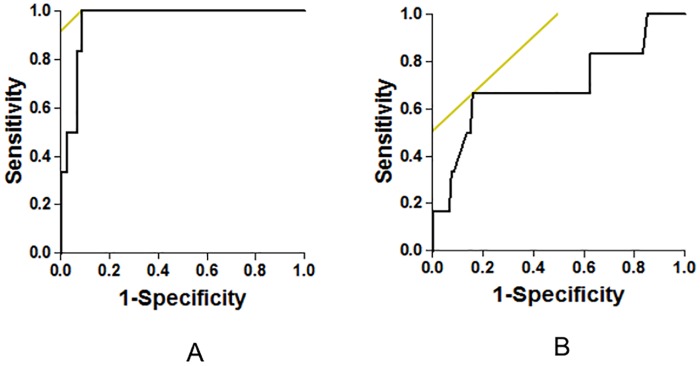
Receiver operative characteristic (ROC) curve analysis of the mean ferritin in the last 12 months (A) and ASDNN (B) for predictors of the cardiac T2* ≤20 ms. ASDNN, the average of the standard deviation of all R-R intervals for all 5-minute segments in the 24-hour recordings.

Correlations between HRV and mean hemoglobin during the last 12 months, mean serum ferritin during the last 12 months, mean serum ferritin during the past 5 years, maximum serum ferritin during the past 5 years, NTBI and cardiac T2* values are summarized in Table 3. Using the multiple linear regression analysis of hemoglobin and all iron markers including serum ferritin, NTBI and cardiac T2* as predictors of HRV, hemoglobin was demonstrated to be the best independent predictor for the all time domains (SDNN, SDANN, ASDNN and rMSSD) of HRV (p = 0.002, p = 0.010, p<0.0001 and p = 0.0001, respectively) and for all frequency domains (LF, HF and LF/HF ratio) of HRV (p<0.0001, p<0.0001 and p = 0.015, respectively). Also, cardiac T2* was a predictor of the ASDNN, LF and HF domains of HRV (p = 0.024, p = 0.025 and p = 0.017, respectively). The scatter pattern of the data showed good correlation between HRV, hemoglobin and cardiac T2* (Fig 1). However, serum ferritin and NTBI were not predictors for HRV. For ROC curve analysis, only the mean ferritin in the last 12 months and ASDNN were predictors of the cardiac T2* ≤20 ms, with area under the ROC curve 0.961 (p<0.0001) and 0.701 (p = 0.05), respectively (Fig 2). Therefore, mean ferritin in the last 12 months of more than 2,800 ng/mL was the best predictor for cardiac T2* ≤20 ms, with 100% sensitivity and 92% specificity. However, depressed ASDNN indicating sympathovagal imbalance was associated with patients who have severe cardiac iron accumulation (cardiac T2* ≤20 ms) and normal left ventricular systolic function.

**Table 3:** The correlations between HRV parameters and mean hemoglobin during the last 12 months, mean serum ferritin during the last 12 months, mean serum ferritin during the past 5 years, maximum serum ferritin during the past 5 years, NTBI and cardiac T2*.

	Mean hemoglobin in the last 12 months	Mean ferritin in the last 12 months	Mean ferritin in the past 5 years	Maximum ferritin in the past 5 years	NTBI	Cardiac T2*
**Time domain**						
SDNN (ms)	0.30┼	-0.17	-0.17	-0.14	-0.21	0.26┼
SDANN (ms)	0.23*	-0.16	-0.14	-0.12	-0.20	0.24*
ASDNN (ms)	0.19	-0.10	-0.02	-0.02	-0.28┼	0.24*
rMSSD (ms)	0.50╪	-0.15	-0.28┼	-0.26┼	-0.29┼	0.17
**Frequency domain**						
LF (ms^2^)	0.41╪	-0.15	-0.14	-0.12	-0.30┼	0.24*
HF (ms^2^)	0.51╪	-0.13	-0.19	-0.17	-0.29┼	0.20*
LF/HF ratio	-0.38╪	0.13	0.26┼	0.26┼	0.18	-0.03

NTBI, non-transferrin bound iron; SDNN, the standard deviation of all normal sinus R-R intervals in the entire 24-hour recording; SDANN, the standard deviation of all average normal sinus R-R intervals for all 5-minute segments in the 24-hour recordings; ASDNN, the average of the standard deviation of all R-R intervals for all 5-minute segments in the 24-hour recordings; rMSSD, the root mean square of the mean of the squared differences of two consecutive R-R intervals in the 24-hour recordings; LF, low frequency power; HF, high frequency power.

* P<0.05

^┼^ P<0.01

^╪^ P<0.001

### Comparison of HRV between thalassemia patients and normal controls

**Fig 3:**
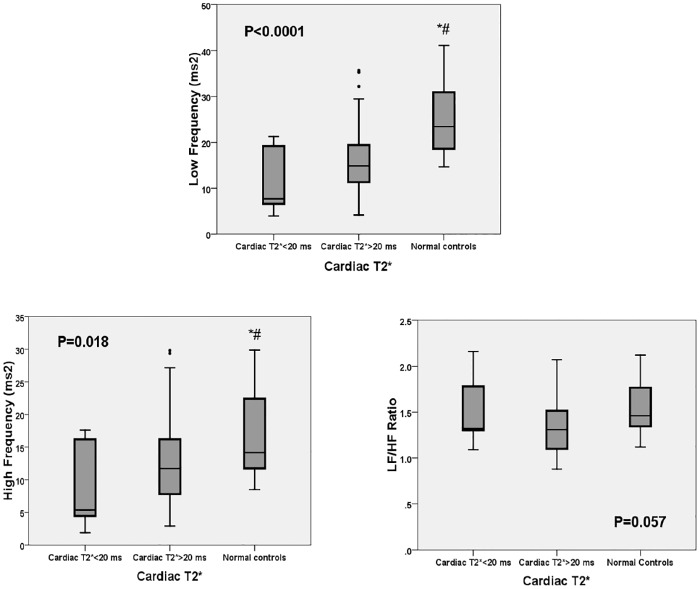
Graphing display comparing the frequency domains of HRV (LF, HF and LF/HF ratio) according to cardiac T2*. LF, low frequency power; HF, high frequency power. *—p<0.05 vs. cardiac T2*<20 ms; #—p<0.05 for cardiac T2*>20 ms vs. normal controls.

**Fig 4:**
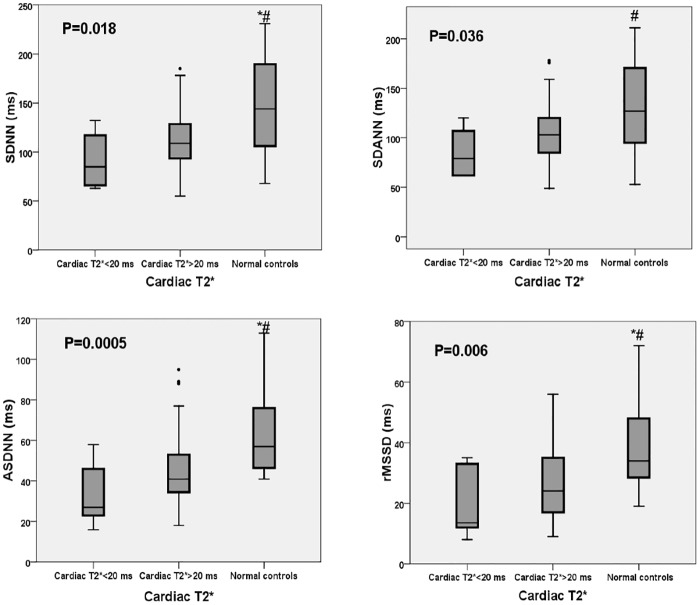
Graphing display comparing the time domains of HRV (SDNN, SDANN, ASDNN and rMSSD) according to cardiac T2*. SDNN, the standard deviation of all normal sinus R-R intervals in the entire 24-hour recording; SDANN, the standard deviation of all average normal sinus R-R intervals for all 5-minute segments in the 24-hour recordings; ASDNN, the average of the standard deviation of all R-R intervals for all 5-minute segments in the 24-hour recordings; rMSSD, the root mean square of the mean of the squared differences of two consecutive R-R intervals in the 24-hour recordings. *—p<0.05 vs. cardiac T2*<20 ms; #—p<0.05 for cardiac T2*>20 ms vs. normal controls.

HRV was compared between patients with cardiac T2* ≤20 ms, cardiac T2* >20 ms, and patients in the control group. The median age in the control group was 21 years (range 20–34 years). The median cardiac T2* in the control group was 36.2 ms (range 29.6–42.2 ms). The LF and HF in the frequency domains of HRV were significantly lower in patients with thalassemia regardless of the cardiac T2* values when compared to the control group (Fig 3). Moreover, all time domains of HRV (SDNN, SDANN, ASDNN and rMSSD) were significantly lower in patients with cardiac T2* ≤20 ms and in those with cardiac T2* >20 ms than in the control group (Fig 4).

## Discussion

Results in this study showed that all time domain parameters of HRV and almost all frequency domain parameters of HRV were significantly lower in all thalassemia patients than those of the controls, and in particular in those patients with cardiac T2* ≤20 ms. Anemia strongly influenced all domains of HRV and it could be a predictor of HRV. After adjusting for anemia, neither serum ferritin nor NTBI impacted the HRV. However cardiac T2* was an independent predictor of HRV, even after adjusting for anemia. Regarding predictors for cardiac T2* ≤20 ms, only ASDNN was an independent HRV predictor of cardiac iron deposition. However, mean ferritin in the last 12 months was the best predictor for the cardiac T2* ≤20 ms with area under the ROC curve of 0.961. Serum ferritin is the most widely used method for assessment of iron overload because of its low cost and availability. A single value might not be reliable because it is influenced by many factors including systemic inflammation, liver disease and ascorbate deficiency [16–17]. The accuracy of serum ferritin can be improved by using the average of serum ferritin over a period of time. All of these findings suggest that the mean value of the annual average of serum ferritin could be useful as an indicator for further evaluation of cardiac iron deposition. However, a single HRV assessment may be used to serve that purpose as well. Our previous study demonstrated that systolic and diastolic left ventricular function was preserved in patients who had a serum ferritin <2500 ng/mL and that diastolic left ventricular dysfunction was present in all patients with serum ferritin >5,000 ng/mL [27]. This study reveals that patients with a mean serum ferritin during the past 5 years >5,000 ng/mL, considered to be severe iron overload, consisted of significantly more homozygous β-thalassemia and splenectomized patients, had lower hemoglobin level, and had more cardiac iron deposits than patients with a mean serum ferritin between 2,500–5,000 ng/mL and <2,500 ng/mL.

When transferrin-binding capacity is exceeded, NTBI appears and enters into vital organs, particularly the heart via the L-type calcium channel, and produces the harmful hydroxyl radical. Excessive free radical generation has an effect on increased lipid peroxidation, impaired Na-K-ATPase activity, increased lysosomal fragility and impaired mitochondrial respiratory chain activity leading to impaired sympathovagal balance, conduction and repolarization disturbance, arrhythmias, and diastolic and systolic myocardial dysfunction [28–30]. All patients with heart disease had presence of NTBI [31]. In our study, all patients had normal left ventricular systolic function. Patients with cardiac T2* ≤20 ms had presence of NTBI. However, NTBI was not a predictor for HRV using multiple linear regression analysis. Also, NTBI was not correlated with the mean ferritin during the past 5 years or with cardiac T2*.

Recently, cardiac T2* MRI has been used as a non-invasive tool for the primary outcome measurement for iron chelation therapy and is used as a gold standard for early detection of iron overload cardiomyopathy [10–17]. Our previous study of 99 patients with non-transfusion dependent thalassemia who had minimal cardiac iron deposit (median T2* of 43.95) and normal left ventricular ejection fraction demonstrated that the LF/HF ratio of HRV was significantly correlated with cardiac T2* (P = 0.026). This finding suggested that HRV could be a tool for early detection of cardiac iron accumulation before myocardial dysfunction [32]. Anderson and colleagues reported that all patients with left ventricular dysfunction had a cardiac T2* ≤20 ms [10]. Using multiple regression analysis of hemoglobin, serum ferritin, NTBI and HRV as possible predictors of cardiac T2*, we found that hemoglobin, mean ferritin in the last 12 months, low frequency, high frequency and ASDNN were predictors of cardiac T2*. For ROC curve analysis, only the mean ferritin in the last 12 months and ASDNN were predictors of cardiac T2* ≤20 ms. ASDNN is the average of the standard deviation of all R-R intervals for all five-minute segments in the 24-hour recordings that estimates short-term components of HRV and reflects sympathetic activity. Depressed ASDNN indicating autonomic imbalance was associated with patients who had severe cardiac iron accumulation (cardiac T2* ≤20 ms) resulting in excessive free radical formation and subsequently increased sympathetic activity. Therefore, ASDNN might be an indicator for early detection of cardiac iron deposition.

One limitation of our study is the small number of patients with severe cardiac iron depositions (cardiac T2* ≤20 ms). However, both domains of HRV were significantly depressed in patients with cardiac T2* ≤20 ms as compared to those with cardiac T2* >20 ms and those in the control group. A long-term prospective study using the 24-hour Holter monitoring for HRV analysis in a greater number of transfusion-dependent β-thalassemia patients with severe cardiac iron accumulation is warranted. The prevalence of abnormal cardiac T2* in this study was lower than those in Europe or the United States, which consisted of primarily of homozygous β-thalassemia patients that have lower residual erythropoietic activity. Therefore, the HRV should be validated in other patient cohorts.

## Conclusions

Hemoglobin and cardiac T2* as predictors for HRV suggest that anemia and cardiac iron deposition influence the cardiac autonomic disturbance. Anemia strongly influenced all domains of HRV and it could be a predictor of HRV. The average of serum ferritin in the last 12 months could be useful as the best indicator for further evaluation of cardiac iron deposition. The ability for serum ferritin to predict cardiac risk is stronger than observed in other thalassemia cohorts and may reflect significant differences in regional genetics, transfusion practices, and availability of iron chelation. HRV might be a stronger predictor of cardiac iron in study populations with lower somatic iron burdens and greater prevalence of cardiac iron deposition.

## Supporting Information

S1 Table Chelation therapy according to the mean serum ferritin during the past 5 years. (DOCX)
