# Supplementary material for: Heart Rate Variability for Early Detection of Cardiac Iron Deposition in Patients with Transfusion-Dependent Thalassemia
Source: PLoS One. 2016 Oct 13;11(10):e0164300. doi: 10.1371/journal.pone.0164300 (PMC5063507; doi:10.1371/journal.pone.0164300)
Supplement: S1 Table — (DOCX) [file pone.0164300.s001.docx]

**S1 Table. Chelation therapy according to the mean serum ferritin during the past 5 years**.

| **Chelation** | **Serum ferritin <2,500 ng/mL (N=64)** | **Serum ferritin 2,500-5,000 ng/mL (N=26)** | **Serum ferritin >5,000 ng/mL (N=11)** | **P value** |
| --- | --- | --- | --- | --- |
| DFO | 3 (5%) | 1 (4%) | 0 | 0.113 |
| DFX | 1 (2%) | 2 (8%) | 0 |  |
| DFP | 19 (30%) | 3 (12%) | 0 |  |
| DFO+DFX | 4 (6%) | 5 (19%) | 1 (9%) |  |
| DFO+DFP | 23 (36%) | 10 (38%) | 6 (55%) |  |
| DFX+DFP | 3 (5%) | 1 (4%) | 0 |  |
| DFO+DFX+DFP | 7 (11%) | 3 (12%) | 4 (36%) |  |

DFO: deferoxamine; DFX: deferasirox; DFP: deferiprone

This table shows the iron chelation therapy that the patients had taken in the past according to the mean serum ferritin during the past 5 years. Patients with serum ferritin >5,000 ng/mL usually received the combination of iron therapy. The selection for iron chelation types depended on the age, severity of iron overload in the liver and heart, other medical diseases, and side effects.
